# Evaluation of the Clinical Performance of the HPV-Risk Assay Using the VALGENT-3 Panel

**DOI:** 10.1128/JCM.01282-17

**Published:** 2017-11-27

**Authors:** N. J. Polman, A. Oštrbenk, L. Xu, P. J. F. Snijders, C. J. L. M. Meijer, M. Poljak, D. A. M. Heideman, M. Arbyn

**Affiliations:** a Department of Pathology, Cancer Center Amsterdam, VU University Medical Center, Amsterdam, The Netherlands; b Institute of Microbiology and Immunology, Faculty of Medicine, University of Ljubljana, Ljubljana, Slovenia; c Unit of Cancer Epidemiology, Belgian Cancer Centre, Scientific Institute of Public Health, Brussels, Belgium; Memorial Sloan Kettering Cancer Center

**Keywords:** cervical cancer screening, human papillomavirus, HPV-Risk assay, Hybrid Capture 2, diagnostic test accuracy, clinical validation, VALGENT, HPV

## Abstract

Human papillomavirus (HPV) testing is increasingly being incorporated into cervical cancer screening. The Validation of HPV Genotyping Tests (VALGENT) is a framework designed to evaluate the clinical performance of various HPV tests relative to that of the validated and accepted comparator test in a formalized and uniform manner. The aim of this study was to evaluate the clinical performance of the HPV-Risk assay with samples from the VALGENT-3 panel and to compare its performance to that of the clinically validated Hybrid Capture 2 assay (HC2). The VALGENT-3 panel comprises 1,300 consecutive samples from women participating in routine cervical cancer screening and is enriched with 300 samples from women with abnormal cytology. DNA was extracted from original ThinPrep PreservCyt medium aliquots, and HPV testing was performed using the HPV-Risk assay by investigators blind to the clinical data. HPV prevalence was analyzed, and the clinical performance of the HPV-Risk assay for the detection of cervical intraepithelial neoplasia grade 3 or worse (CIN3+) and CIN2 or worse (CIN2+) relative to the performance of HC2 was assessed. The sensitivity of the HPV-Risk assay for the detection of CIN3+ was similar to that of HC2 (relative sensitivity, 1.00; 95% confidence interval [CI], 0.95 to 1.05; *P* = 1.000), but the specificity of the HPV-Risk assay was significantly higher than that of HC2 (relative specificity, 1.02; 95% CI, 1.01 to 1.04; *P* < 0.001). For the detection of CIN2+, similar results were obtained, with the relative sensitivity being 0.98 (95% CI, 0.93 to 1.02; *P* = 0.257) and the relative specificity being 1.02 (95% CI, 1.01 to 1.03; *P* < 0.001). The performance of the HPV-Risk assay for the detection of CIN3+ and CIN2+ was noninferior to that of HC2, with all *P* values being ≤0.006. In conclusion, the HPV-Risk assay demonstrated noninferiority to the clinically validated HC2 by the use of samples from the VALGENT-3 panel for test validation and comparison.

## INTRODUCTION

Screening with human papillomavirus (HPV) testing provides better protection against cervical cancer and high-grade cervical intraepithelial neoplasia (CIN) than cytology-based screening (1, 2). Consequently, HPV testing is increasingly being incorporated into clinical protocols and cervical cancer screening guidelines. A substantial number of different HPV tests is currently available on the market (3); however, clinical validation of a test is required before its use for screening purposes.

The international validation guidelines described by Meijer et al. (4) have been used for the translation of HPV testing into clinical practice by setting standards for test performance and characteristics. However, the proper validation of HPV tests can be problematic due to the difficulties with obtaining an appropriate set of clinical specimens. The Validation of HPV Genotyping Tests (VALGENT) framework facilitates the comparison and validation of HPV tests by providing a set of samples obtained from women attending routine screening enriched with cytologically abnormal samples (5–9). In order to allow comparison with other HPV tests, each VALGENT panel includes a comparator assay that is clinically validated for cervical cancer screening purposes.

The HPV-Risk assay is a real-time PCR-based assay that targets the E7 region of 15 (probable) high-risk HPV types and enables partial genotyping for HPV16 and HPV18. A previous evaluation of the HPV-Risk assay relative to the clinically validated comparator GP5+/6+-PCR showed that its clinical performance and reproducibility met the international validation criteria for HPV tests for use in cervical cancer screening (10).

This study was performed to evaluate the clinical performance of the HPV-Risk assay for detection of high-grade CIN using the VALGENT-3 panel and to compare its performance to that of the clinically validated Hybrid Capture 2 assay (HC2) as a comparator test (4, 11).

## RESULTS

### HPV-Risk assay results.

Of the 1,600 samples tested by the HPV-Risk assay, 30 (1.9%) samples were considered invalid for PCR amplification and were excluded from further analysis. HPV-Risk assay and HC2 results are shown in Table 1. Of the 1,570 (98.1%) valid results, 1,272 were from the screening population (including 9 CIN grade 2 [CIN2] and 11 CIN grade 3 or worse [CIN3+] cases) and 298 were from the enrichment population (including 36 CIN2 and 71 CIN3+ cases). Of 1,272 women in the screening population with valid HPV-Risk assay results, 133 (10.5%) women tested HPV positive with the HPV-Risk assay. The prevalence of HPV16 and HPV18 was 2.6% (33/1,272) and 0.9% (12/1,272), respectively. Of 298 women in the enrichment population with valid HPV-Risk assay results, 205 (68.8%) women tested HPV positive. The prevalence of HPV16 and HPV18 was 27.2% (81/298) and 7.4% (22/298), respectively. For comparison, 159 (12.5%) women in the screening population and 214 (71.8%) women in the enrichment population tested HPV positive with HC2.

**TABLE 1:** HPV-Risk assay and HC2 results for screening and enrichment populations of VALGENT-3 panel^*a*^

Assay and result	Screening population		Enrichment population		Total	
	*n*	%	*n*	%	*n*	%
HPV-Risk assay						
HPV positive	133	10.5	205	68.8	338	21.5
HPV16 positive	33	2.6	81	27.2	114	7.3
HPV18 positive	12	0.9	22	7.4	34	2.2
HPV negative	1,139	89.5	93	31.2	1,232	78.5
HC2						
HPV positive	159	12.5	214	71.8	373	23.8
HPV-negative	1,113	87.5	84	28.2	1,197	76.2
Total	1,272	100.0	298	100.0	1,570	100.0

a HPV, human papillomavirus; *n*, number of cases.

### Clinical performance of the HPV-Risk assay.

The absolute sensitivity and specificity of the HPV-Risk assay for the detection of CIN3+ and CIN grade 2 or worse (CIN2+) are shown in Table 2, both for the total study group (*n* = 1,316) and for women ≥30 years old (*n* = 1,088). Within the total study group, the sensitivity of the HPV-Risk assay for the detection of CIN3+ was 97.6% (95% confidence interval [CI], 91.5 to 99.7%) and the specificity was 89.0% (95% CI, 87.1 to 90.7%). For the detection of CIN2+, the sensitivity and specificity were 93.7% (95% CI, 88.0 to 97.2%) and 91.8% (95% CI, 90.1 to 93.3%), respectively. For women aged ≥30 years (*n* = 1,088), the sensitivity of the HPV-Risk assay for the detection of CIN3+ was 97.0% (95% CI, 89.5 to 99.6%) and the specificity was 91.8% (95% CI, 89.9 to 93.4%). For the detection of CIN2+ among women aged ≥30 years, the sensitivity and specificity of the HPV-Risk assay were 92.9% (95% CI, 85.8 to 97.1%) and 94.2% (95% CI, 92.6 to 95.6%), respectively.

**TABLE 2:** Sensitivity and specificity of HPV-Risk assay and HC2 for detection of CIN3+ and CIN2+^*a*^

Assay, group studied, and CIN grade	Sensitivity			Specificity		
	*n*/*N*	%	95% CI	*n*/*N*	%	95% CI
HPV-Risk assay						
Total group						
CIN3+	80/82	97.6	(91.5–99.7)	1,098/1,234	89.0	(87.1–90.7)
CIN2+	119/127	93.7	(88.0–97.2)	1,092/1,189	91.8	(90.1–93.3)
Women ≥30 yr old						
CIN3+	64/66	97.0	(89.5–99.6)	938/1,022	91.8	(89.9–93.4)
CIN2+	91/98	92.9	(85.8–97.1)	933/990	94.2	(92.6–95.6)
HC2						
Total study group						
CIN3+	80/82	97.6	(91.5–99.7)	1,072/1,234	86.9	(84.6–88.7)
CIN2+	122/127	96.1	(91.1–98.7)	1,069/1,189	89.9	(88.1–88.7)
Women ≥30 yr old						
CIN3+	64/66	97.0	(89.5–99.6)	918/1,022	89.8	(87.8–91.6)
CIN2+	94/98	95.9	(89.9–98.9)	916/990	92.5	(90.7–94.1)

a HPV, human papillomavirus; CIN3+, cervical intraepithelial neoplasia grade 3 or worse; CIN2+, cervical intraepithelial neoplasia grade 2 or worse; *n*, number of cases; *N*, total number of cases; CI, confidence interval.

The absolute sensitivities and specificities of HC2 for the detection of CIN3 and CIN2+ are shown in Table 2. Cross-tabulations of the results of the HPV-Risk assay and HC2 are shown in Table 3. The corresponding relative sensitivities of the HPV-Risk assay versus those of HC2 for the detection of CIN3+ and CIN2+ as well as the relative specificities of the HPV-Risk assay for the detection of cervical intraepithelial neoplasia grade 1 or lower (≤CIN1) are shown in Table 4. The sensitivity of the HPV-Risk assay for the detection of CIN3+ was the same as that of HC2 (97.6% versus 97.6%; relative sensitivity, 1.00; 95% CI, 0.95 to 1.05; *P* value by the McNemar test [*P*_McN_] = 1.000), but its specificity was significantly higher (89.0% versus 86.9%; relative specificity, 1.02; 95% CI, 1.01 to 1.04; *P*_McN_ < 0.001). Similar results were obtained for the detection of CIN2+ (i.e., a sensitivity of 93.7% versus 96.1% [relative sensitivity, 0.98; 95% CI, 0.93 to 1.02; *P*_McN_ = 0.257] and a specificity of 91.8% versus 89.9% [relative specificity, 1.02; 95% CI, 1.01 to 1.03; *P*_McN_ < 0.001]). The performance of the HPV-Risk assay was clinically noninferior to that of HC2 with respect to sensitivity and specificity for the detection of CIN3+ (*P* value for the noninferiority of the HPV-Risk assay to HC2 [*P*_ni_] = 0.003 and *P*_ni_ < 0.001, respectively) and also for CIN2+ (*P*_ni_ = 0.006 and *P*_ni_ < 0.001, respectively).

**TABLE 3:** Comparison of HPV-Risk assay and HC2 for HPV detection stratified by clinical outcome^*a*^

CIN grade	HPV-Risk assay result	No. of women with the indicated HC2 result					
		Total study group			Women ≥30 yr old		
		Positive	Negative	Total	Positive	Negative	Total
CIN3+	Positive	78	2	80	62	2	64
	Negative	2	0	2	2	0	2
	Total	80	2	82	64	2	66
CIN2+	Positive	117	2	119	89	2	91
	Negative	5	3	8	5	2	7
	Total	122	5	127	94	4	98
≤CIN1	Positive	87	10	97	47	10	57
	Negative	33	1,059	1,092	27	906	933
	Total	120	1,069	1,189	74	916	990

a HPV, human papillomavirus; CIN3+, cervical intraepithelial neoplasia grade 3 or worse; CIN2+, cervical intraepithelial neoplasia grade 2 or worse; ≤CIN1, cervical intraepithelial neoplasia grade 1 or lower, i.e., women with two consecutive negative cytology results (control group).

**TABLE 4:** Relative sensitivities for detection of CIN3+ and CIN2+ and relative specificities for detection of ≤CIN1 of HPV-Risk assay versus HC2^*a*^

Group studied and CIN grade	Relative sensitivity	Relative specificity	*P*_McN_	*P*_ni_
Women of all ages				
CIN3+	1.00 (0.95–1.05)^*b*^		1.000	0.003
CIN2+	0.98 (0.93–1.02)		0.257	0.006
≤CIN1		1.02 (1.01–1.03)	<0.001	<0.001
Women ≥30 yr old				
CIN3+	1.00 (0.94–1.06)		1.000	0.007
CIN2+	0.97 (0.92–1.02)		0.257	0.010
≤CIN1		1.02 (1.01–1.03)	0.005	<0.001

a HPV, human papillomavirus; CIN3+, cervical intraepithelial neoplasia grade 3 or worse; CIN2+, cervical intraepithelial neoplasia grade 2 or worse; ≤CIN1, cervical intraepithelial neoplasia grade 1 or lower, i.e., women with two consecutive negative cytology results (control group); *P*_McN_, *P* value by the McNemar test; *P*_ni_, *P* value for the noninferiority of HPV-Risk assay to HC2.

b Values in parentheses are 95% confidence intervals.

When we performed the analyses for women aged ≥30 years, results similar to those obtained for the total study group were obtained (Table 4). Relative sensitivity and specificity for the detection of CIN3+ in women aged ≥30 years were 1.00 (95% CI, 0.94 to 1.06; *P*_McN_ = 1.000) and 1.02 (95% CI, 1.01 to 1.04; *P*_McN_ = 0.002), respectively. Relative sensitivity and specificity for the detection of CIN2+ were 0.97 (95% CI, 0.92 to 1.02; *P*_McN_ = 0.257) and 1.02 (95% CI, 1.01 to 1.03; *P*_McN_ = 0.005), respectively. In women aged ≥30 years, the performance of the HPV-Risk assay was clinically noninferior to that of HC2 with respect to the sensitivity and specificity for the detection of CIN3+ (*P*_ni_ = 0.007 and *P*_ni_ < 0.001, respectively) and CIN2+ (*P*_ni_ = 0.010 and *P*_ni_ < 0.001, respectively).

## DISCUSSION

In this study, we evaluated the clinical performance of the HPV-Risk assay for the detection of high-grade CIN and compared its performance to that of a clinically validated test (HC2) using samples from the VALGENT-3 panel. The results of this study show that the sensitivity of the HPV-Risk assay for the detection of CIN3+ and CIN2+ was comparable to that of HC2 and that it had a significantly higher specificity, both in the total study group and in women aged ≥30 years. The HPV-Risk assay showed a noninferior clinical performance compared to that of HC2.

Clinical validation studies of HPV tests are crucial because the HPV assays that are used for primary cervical cancer screening must ensure an optimal distinction between HPV infections associated with CIN2+/3+ and clinically irrelevant transient HPV infections (4). The current study, as an independent clinical validation in a different study cohort with a different clinically validated comparator test (HC2), further supports previously published clinical validation data for the HPV-Risk assay (10).

An advantage of the HPV-Risk assay, in comparison with other commercially available HPV assays, is that no specific laboratory equipment is needed since the HPV-Risk assay can run on different real-time PCR platforms and is compatible with various collection media and different DNA extraction procedures. Furthermore, the HPV-Risk assay can be performed reliably on self-sampled material, both lavage-based and brush-based material, in addition to physician-taken cervical scrape samples (10, 12, 13). The turnaround time of the HPV-Risk PCR method is about 1 h. However, the turnaround time of the total procedure, from sample collection to HPV test result, depends on the duration of the DNA extraction method that is used. Finally, the HPV-Risk assay targets a conserved region within the E7 open reading frame of the respective high-risk HPV types. A recent study has shown that the E7 region of HPV16, in contrast to most other open reading frames, is highly conserved in cervical cancer and precancer (14). Therefore, the use of the HPV-Risk assay might be associated with a lower risk of nondetection of cervical cancer and precancer caused by HPV16 than for assays that target other open reading frames which are more variable in cancer and precancer.

The strength of this study is the large sample set from the VALGENT-3 framework, which makes it possible to evaluate the clinical performance of various HPV assays compared to that of the validated and accepted comparator test (HC2) in a formalized and uniform manner. In addition, a network of test comparisons, which can later be pooled in multiple-testing meta-analysis, can be developed (4, 5, 11). Another strength is that testing by the HPV-Risk assay was performed by investigators completely blind to the clinical data and cytology findings. Furthermore, in accordance with the VALGENT framework criteria, aliquots of original, nonisolated cervical scrape material were sent to the test laboratory; therefore, not only the assay itself but also the entire laboratory process from sample processing to the HPV test result was evaluated. Finally, in VALGENT-3, women were managed on the basis of cytology and HPV screening test results, whereas in a previous VALGENT round, VALGENT-2, detection of (pre-)cancerous lesions was triggered only through cytology (6–9). Therefore, the VALGENT-2 panel might have included a circumstantial advantage for sensitivity and a disadvantage for specificity. On the other hand, a relatively short active follow-up period in VALGENT-3 (12 to 48 months) may be considered a limitation. However, since HC2 was used as a comparator test that was validated through randomized trials with follow-up over 8 years, its cross-sectional accuracy is considered sufficient for validation. Nevertheless, VALGENT-3 results will soon most likely be linked to the outcomes of subsequent cervical cancer screening rounds (continuous passive follow-up through the Slovenian National Cervical Cancer Screening Registry and National Cancer Registry), which will provide information on long-term safety.

In conclusion, the HPV-Risk assay has a high sensitivity and a high specificity for the detection of CIN3+ and CIN2+ and has a demonstrated noninferiority to the clinically validated HC2. Results from this study provide additional evidence that the HPV-Risk assay can be considered clinically validated and therefore can be safely used for primary cervical cancer screening.

## MATERIALS AND METHODS

### VALGENT-3 panel.

The VALGENT-3 panel was collated in Slovenia (5). Ethical approval was obtained from the Medical Ethics Committee of the Republic of Slovenia (consent numbers 83/11/09 and 109/08/12). The panel is standardized, comprising 1,300 consecutive samples from women participating in the organized national cervical cancer screening program with more than 70% coverage (screening population), enriched with 300 samples from women with abnormal cytology (enrichment population). The characteristics of the two study populations are shown in Table 5.

**TABLE 5:** Characteristics of screening and enrichment populations of the VALGENT-3 panel^*a*^

Characteristic	Value for:		
	Screening population	Enrichment population	Total
Mean (range) age (yr)	39 (21–64)	37 (20–77)	39 (20–77)
No. (%) of women age:			
≥30 yr	1,085 (83.5)	221 (73.7)	1,306 (81.6)
<30 yr	215 (16.5)	79 (26.3)	294 (18.4)
No. (%) of women with the following cytology:			
Normal cytology	1,238 (95.2)	0 (0.0)	1,238 (77.4)
ASC-US	31 (2.4)	100 (33.3)	131 (8.2)
LSIL	13 (1.0)	100 (33.3)	113 (7.1)
ASC-H	3 (0.2)	0 (0.0)	3 (0.2)
HSIL	14 (1.1)	100 (33.3)	114 (7.1)
AGC	1 (0.1)	0 (0.0)	1 (0.1)
No. (%) of women with the following histology:			
No histology	1,266 (97.4)	80 (26.7)	1,346 (84.1)
Normal histology	4 (0.3)	73 (24.3)	77 (4.8)
CIN1	10 (0.8)	40 (13.3)	50 (3.1)
CIN2	9 (0.7)	36 (12.0)	45 (2.8)
CIN3 (including CIS)	11 (0.8)	69 (23.0)	80 (5.0)
SCC	0 (0.0)	1 (0.3)	1 (0.1)
Adenocarcinoma	0 (0.0)	1 (0.3)	1 (0.1)
Total	1,300 (100.0)	300 (100.0)	1,600 (100.0)

a The screening population included 1,300 subjects, and the enrichment population included 300 subjects. ASC-US, atypical squamous cells of undetermined significance; LSIL, low-grade squamous intraepithelial lesion; ASC-H, atypical squamous cells (the possibility of a high-grade squamous intraepithelial lesion cannot be excluded); HSIL, high-grade squamous intraepithelial lesion; AGC, atypical glandular cells; CIN, cervical intraepithelial neoplasia; CIS, carcinoma *in situ*; SCC, squamous cell carcinoma.

Cervical scrape samples from the screening population were collected from December 2009 to August 2010, and samples from the enrichment population were collected from January 2014 to May 2015. All samples were collected in ThinPrep PreservCyt medium (Hologic, Bedford MA, USA) according to European Union guidelines (15). Samples were transported to the University of Ljubljana (Ljubljana, Slovenia) weekly. Several aliquots were prepared from each cervical specimen. One aliquot was used for HC2 testing, and the remaining aliquots were immediately stored at −70°C and later sent to participating laboratories for HPV testing. HC2 was performed according to the manufacturer's instructions (Qiagen, Gaithersburg MD, USA) at the University of Ljubljana (Ljubljana, Slovenia) during the period from December 2009 to August 2010 (16). HC2 testing was performed within 2 weeks after sample collection. Briefly, HC2 uses a cocktail of captured RNA probes to detect 13 high-risk HPV types (i.e., HPV16, -18, -31, -33, -35, -39, -45, -51, -52, -56, -58, -59, and -68). The probes hybridize with viral DNA, and the captured HPV RNA/DNA hybrids are identified by a secondary capture system that yields a light signal. The intensity of the light signal can be semiquantitatively related to the viral load (17–19).

Management of the women was based on the results of cytology and HPV testing (by HC2 and the Abbott RealTime High Risk HPV test), as previously described in detail (16). Women with atypical-squamous-cell (the possibility of a high-grade squamous intraepithelial lesion cannot be excluded) (ASC-H) or atypical-glandular-cell (AGC) cytology or worse were invited for colposcopy, according to the criteria of the Slovenian National Cervical Cancer Screening Program and European Union guidelines for quality assurance for cervical cancer screening (3, 16, 20). In addition, women who tested positive for HPV16 or HPV18 were invited for colposcopy irrespective of their cytology result. Women who tested HPV-non-16/18 positive were invited for either colposcopy or a control visit after 6 to 12 months, at the physician's discretion. During colposcopy, biopsy specimens were taken from any regions suspicious for CIN. Histological samples were classified as CIN grade 0, 1, 2, or 3 or as invasive cancer (21, 22).

### HPV-Risk assay.

For testing with the HPV-Risk assay, ThinPrep aliquots were transported to the VU University Medical Center (Amsterdam, The Netherlands) in June 2016. Extraction of DNA from the ThinPrep aliquots was performed using an automated extraction system (Macherey-Nagel, Duren, Germany) during the period from August to December 2016, with subsequent HPV-Risk assay testing being performed according to the manufacturer's instructions (Self-screen BV, Amsterdam, The Netherlands). Accordingly, the time between sample collection and HPV-Risk assay testing was approximately 7 years. The HPV-risk assay targets the E7 region of 15 (probably) high-risk HPV types. Detection is based on hydrolysis probes with 3 spectrally unique fluorescent dyes, providing genotype information for HPV16 and HPV18 and a pooled detection of the 13 other HPV types (i.e., HPV31, -33, -35, -39, -45, -51, -52, -56, -58, -59, -66, -67, and -68) (10). The fourth channel enables detection of the human β-globin gene using a probe labeled with a different fluorescent dye, intended for assessment of the sample quality control (internal control) and potential inhibition. Samples were considered HPV positive when the threshold cycle (*C_T_*) values for HPV16, HPV18, and/or other HPV types were ≤36. If no HPV signals were obtained and the *C_T_* value for the β-globin target was ≤33, samples were considered HPV negative. Samples were considered invalid when the *C_T_* value for HPV was >36 and that for β-globin was >33.

All HPV testing was performed by investigators blind to the clinical data and cytology findings. Results obtained by testing by both HPV assays were sent to the Unit of Cancer Epidemiology, Scientific Institute of Public Health (Brussels, Belgium), where analysis and evaluation of the data were performed.

### Statistical analysis.

On the basis of the HPV-Risk assay results, the overall prevalence of high-risk HPV infection and the type-specific prevalence of HPV16 and HPV18 infection were assessed in the screening population of 1,300 women. For clinical validation, women with histologically confirmed CIN2, CIN3, and cervical cancer within 48 months after sample collection were classified as having high-grade disease (disease group). Women with two consecutive negative cytology results (at enrollment and at 12 to 48 months of follow-up) were classified as having no evidence of disease (control group). The clinical performance of the HPV-Risk assay for the detection of both CIN3+ (primary endpoint) and CIN2+ (secondary endpoint) was assessed in the total study group and in women aged ≥30 years, and the clinical performance of the HPV-Risk assay was compared to the clinical performance of HC2 as the standard comparator assay. Sensitivity and specificity were calculated from cross-tabulation of the test results with exact 95% confidence intervals (CIs).

Relative sensitivities (ratios of the sensitivity of one test to the sensitivity of another test) and relative specificities (ratios of the specificity of one test to the sensitivity of another test) with 95% CIs were calculated. The McNemar (McN) test was applied to assess differences between matched proportions. A *P*_McN_ value of >0.05 indicated that the sensitivity or specificity of the HPV-Risk assay was not significantly different from that of HC2. Finally, the noninferiority (ni) of the HPV-Risk assay to HC2 was assessed, according to the international validation criteria (4, 23). In order to demonstrate noninferiority, the sensitivity of the HPV-Risk assay had to be at least 90% and the specificity had to be at least 98% compared to the results of HC2. A *P*_ni_ value of <0.05 indicated that the sensitivity or specificity of the HPV-Risk assay was not significantly lower than that of HC2.
